# Select amino acids in DGCR8 are essential for the UGU-pri-miRNA interaction and processing

**DOI:** 10.1038/s42003-020-1071-5

**Published:** 2020-07-03

**Authors:** Thi Lieu Dang, Cong Truc Le, Minh Ngoc Le, Trung Duc Nguyen, Thuy Linh Nguyen, Sheng Bao, Shaohua Li, Tuan Anh Nguyen

**Affiliations:** Division of Life Science, The Hong Kong University of Science & Technology, Hong Kong, China

**Keywords:** Molecular biology, Biochemistry

## Abstract

Microprocessor, composed of DROSHA and DGCR8, processes primary microRNAs (pri-miRNAs) in miRNA biogenesis. Its cleavage efficiency and accuracy are enhanced because DGCR8 interacts with the apical UGU motif of pri-miRNAs. However, the mechanism and influence of DGCR8–UGU interaction on cellular miRNA expression are still elusive. In this study, we demonstrated that Rhed (i.e., the RNA-binding heme domain, amino acids 285–478) of DGCR8 interacts with UGU. In addition, we identified three amino acids 461–463 in Rhed, which are critical for the UGU interaction and essential for Microprocessor to accurately and efficiently process UGU-pri-miRNAs in vitro and UGU-miRNA expression in human cells. Furthermore, we found that within the DGCR8 dimer, the amino acids 461–463 from one monomer are capable of discriminating between UGU- and noUGU-pri-miRNAs. Our findings improve the current understanding of the substrate-recognizing mechanism of DGCR8 and implicate the roles of this recognition in differentiating miRNA expression in human cells.

## Abstract (web summary)

Thi Lieu Dang et al. study the mechanisms for the interaction between DGCR8 and the apical UGU motif of pri-miRNAs. They demonstrate that three amino acids in the Rhed domain of DGCR8 are critical to recognize and interact with UGU and to process pri-miRNAs. They further show amino acids 461–463 in one of the DGCR8 dimer are necessary to distinguish UGU- and noUGU-primiRNAs.

## Introduction

MicroRNAs (miRNAs) are small single-stranded RNAs (ssRNAs) of 21–22 nucleotides (nt) in humans. miRNAs regulate gene expression by either triggering mRNA degradation or/and inhibiting mRNA translation. miRNAs function in numerous vital cellular processes and are associated with various human diseases^1,2^. In humans, approximately 2500 miRNAs have been identified^3^. Most miRNAs are synthesized via a canonical miRNA biogenesis pathway, which involves two human RNase III enzymes, DROSHA and DICER^2^. Initially, transcripts of a miRNA precursor, called primary miRNAs (pri-miRNAs) are produced in the nucleus by RNA polymerase II. These pri-miRNAs are then cleaved by the human Microprocessor complex (which consists of DROSHA and its cofactor, DGCR8), to generate stem–loop-containing RNA molecules, called pre-miRNAs. Pre-miRNAs are subsequently exported to the cytoplasm where they are further processed by DICER, giving rise to RNA duplexes. The RNA duplexes become associated with Ago proteins, such that one of the strands of the duplex is retained in Ago, while the other is discarded^1,4,5^. The Ago-associated ssRNAs function as mature miRNAs to successively target mRNAs and silence gene expression^1,4–7^.

DROSHA is the catalytic subunit of the Microprocessor complex, while DGCR8 is its cofactor^8–18^. The major substrates of Microprocessor in cells are pri-miRNAs. Their stem is usually an imperfect 35 base pairs (bp), and the loop, also called an apical loop, varies in length^2,15^. The stem length and loop size are important for pri-miRNA processing^19–21^. The apical loop connects to one end of the stem via the apical junction, whereas two ssRNA segments, namely the 5p- and 3p-basal segments, flank the other end, making a basal junction^2,15^. Pri-miRNAs contain several RNA elements, such as UG, UGU, mGHG, CNNC, seedMW, and midMW, which are critical for the efficiency and accuracy of pri-miRNA processing by Microprocessor^8,10,17,22–28^. The recent report demonstrated that the internal loop in the lower stem of pri-miRNAs facilitates the single cleavage of pri-miRNAs on the 5p-strand and reduces the double cleavages of Microprocessor, thereby controlling miRNA biogenesis^29^.

In the Microprocessor complex, DROSHA and DGCR8 are responsible for recognizing and interacting with different RNA elements of pri-miRNAs. DROSHA interacts with the basal junction and the basal UG motif of pri-miRNAs and executes productive cleavages to generate pre-miRNAs^8,10,30,31^. DROSHA also interacts via its double-stranded RNA-binding domain (dsRBD), with the mGHG motif located in the lower stem of pri-miRNAs, and this influences the accuracy of DROSHA cleavage^22,25,30,31^. DGCR8 is a multi-domain protein, which helps DROSHA cleave pri-miRNAs precisely and efficiently. The C-terminal tail region of DGCR8 (CTT) interacts with and solubilizes DROSHA, which activates its enzymatic activity^10,26,32^. In addition, the dsRBDs of DGCR8 enhance the RNA-binding affinity of the complex and thus stimulate the enzymatic efficiency^10,32^. The Rhed domain (i.e., the RNA-binding heme domain, amino acids 285–478) of DGCR8 is responsible for its ability to form dimers^11^. This domain also has an RNA-binding ability and associates with the small molecule, hemin^11,33,34^. The dimerization, RNA-binding ability, and hemin-association ability of Rhed are all essential for pri-miRNA processing^10,11,26,33,35^. DGCR8 interacts with the apical loop and its UGU motif. This DGCR8–UGU interaction is vital for pri-miRNA processing on UGU-containing pri-miRNAs in vitro^10,26^. In addition, the DGCR8–UGU interaction is under-regulated by some factors, such as hemin and RNA polymerase II, which differentiate miRNA expression^26,36^. Therefore, having a better understanding of the DGCR8–UGU interaction is essential for determining the role of Microprocessor in pri-miRNA processing. In a previous study, we showed that the Rhed-containing fragments of DGCR8 recognize the UGU motif, whereas the fragments lacking Rhed fail to do so^10^. This suggests that Rhed alone might be able to recognize the UGU motif. However, there is still no direct evidence to indicate if (and how) Rhed recognizes and interacts with the UGU motif, and how this interaction influences both pri-miRNA processing in vitro and the expression of miRNA in cells. Besides, it is not clear how each subunit in the DGCR8 dimer contributes to the UGU-interaction.

In this study, we utilized the in vitro pri-miRNA processing system along with electrophoresis mobility shift assays (EMSAs) to explore the UGU-recognizing mechanism of DGCR8. We demonstrated that Rhed alone is sufficient to interact with the UGU motif, and identified amino acids 461–463 in Rhed, as being responsible for UGU-recognition. We also demonstrated that these amino acids are important for pri-miRNA processing in vitro and cellular miRNA expression, especially for UGU-containing miRNAs. Also, we found that amino acids 461–463 in either of the monomers of the DGCR8 dimer are necessary for the UGU-interaction. Our findings advance our understanding of the role of the DGCR8–RNA interaction in the pri-miRNA processing mechanism and miRNA biogenesis.

## Results

### Rhed directly recognizes the UGU motif

**Fig. 1:**
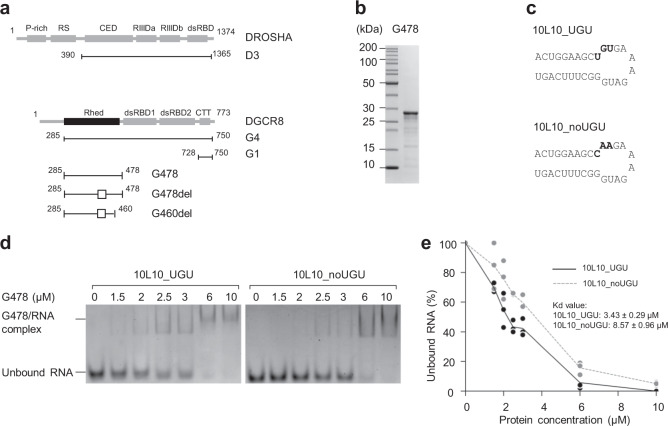
Rhed recognizes the UGU motif. **a** The protein constructs used in this study. For each construct, the first and last amino acid residue positions are shown. The box marks the deleted regions from amino acids 370–429. P-rich: proline-rich domain, RS: arginine/serine-rich domain, CED: central domain, RIIIDa and RIIIDb: RNase III domains, dsRBD: dsRNA-binding domain, Rhed: RNA-binding heme domain, and CTT: C-terminal tail region. **b** SDS-PAGE to show the purified G478 protein. **c** Structure diagrams and ribonucleotide sequences of 10L10_UGU and 10L10_noUGU. **d** The EMSAs for G478. Various amounts of G478 (ranging from 0 to 10 μM) were mixed with 1 μM of either 10L10_UGU or 10L10_noUGU in a 10 μL reaction solution. The reaction mixture was run on a 4% native PAGE gel. **e** Quantification of the EMSA data shown in (**d**). The density of each RNA band was measured using Image Lab 6.0 (Bio-Rad), and the results were obtained from three independent experiments.

A previous study suggested that the DGCR8 dimer interacts with the UGU motif of pri-miRNAs^10^. The purified DGCR8 dimer, comprising two G4 fragments (Fig. 1a), interacted with UGU and enhanced the specific cleavage of Microprocessor. The G4 monomer was shown to have a weaker UGU-interaction than the G4 dimer. In contrast, a shorter DGCR8 fragment (G3) containing the CTT domain and two dsRBDs, did not efficiently bind the UGU motif^10^. These suggest that the Rhed dimer might be responsible for UGU-recognition. We first estimated an RNA-binding affinity of the DGCR8 dimer with pri-mir-30a_UGU and pri-mir-30a_noUGU using the EMSAs. However, we could not observe the difference in the RNA-binding affinity of DGCR8 with these two pri-miRNAs. Perhaps, four dsRBDs of the DGCR8 dimer interacted with the double-stranded RNA (dsRNA) region of pri-miRNA so strongly that the defect caused by noUGU was not seen (Supplementary Fig. 1a–c). We then expressed and purified only a Rhed dimer of DGCR8 (G478, amino acids 285–478) from *Escherichia coli* (Fig. 1a, b) and examined its interaction with pri-mir-30a_UGU and pri-mir-30a_noUGU using the EMSAs. Using this approach, we could detect a slight difference in the RNA-binding affinity of G478 with pri-mir-30a_UGU and pri-mir-30a_noUGU (Supplementary Fig. 1d, compare lanes 2, 3 and 7, 8). Since pri-mir-30a_UGU contained the long stem, apical loop, and basal segments, multiple G478 molecules could bind to one pri-mir-30a_UGU. Therefore, we decided to use a short stem–loop RNA with or without the UGU motif for further investigation (Fig. 1c).

We confirmed the ability of Rhed to associate with hemin by measuring the absorbance of the purified proteins at a wavelength of 450 nm^11^ using Agilent 1200 high-performance liquid chromatography (HPLC; Supplementary Fig. 1e). We also assessed the dimeric state of the purified Rhed protein by estimating its molecular mass using a Multi-Angle Light Scattering detector (miniDAWN TREOS, Wyatt Technology Corporation). The estimated molecular mass of G478 was ~45 kDa, indicating that the purified G478 protein existed in a dimeric form with a theoretical molecular mass of ~44 kDa (Supplementary Fig. 1f). The RNA-binding affinity of G478 with these 10L10 substrates was estimated using the EMSAs, as described in “Methods”. The results in Fig. 1d, e show that G478 interacted with 10L10_UGU (*K*_d_ = 3.43 ± 0.29 μM) much stronger than it did with 10L10_noUGU (*K*_d_ = 8.57 ± 0.96 μM), and this resulted in a higher level of RNA shift in the EMSA gels. These data indicate that the G478 dimer sufficiently recognizes and interacts with the UGU motif.

### Amino acids 460–478 are critical for RNA-binding

**Fig. 2:**
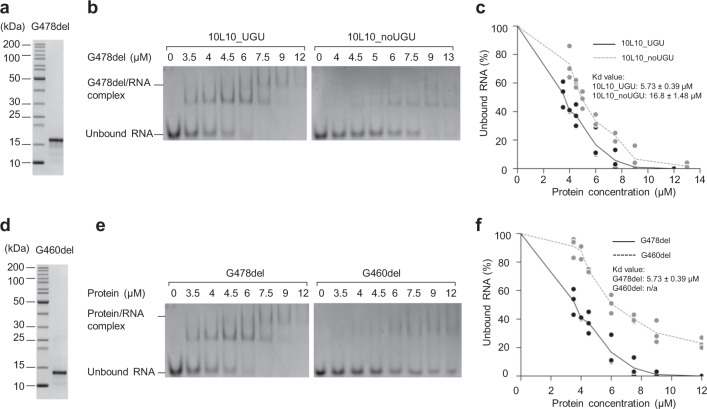
Amino acids 460–478 of Rhed are critical for hemin binding and RNA interaction. **a**, **d** Purified G478del (**a**) and G460del (**d**) proteins analyzed by SDS-PAGE. **b**, **e** The EMSAs for G478del (**b**) and G460del (**e**). Various amounts of G478del (**b**) or G460del (**e**) (ranging from 0 to 13 μM) were mixed with 1 μM of 10L10_UGU or 10L10_noUGU in a 10 μL reaction solution. **c**, **f** Quantification of the EMSA data in (**b**) and (**e**) for G478del and G460del, respectively. The density of each RNA band on the gel was measured using Image Lab 6.0 (Bio-Rad), and the results were obtained from three independent experiments.

To determine which of the amino acids of Rhed might be responsible for UGU-recognition, we generated various shorter fragments of DGCR8 and tested their RNA-binding capacity. Since the region spanning amino acids 285–370 is essential for dimerization^11,34^, we hypothesized that the RNA-binding affinity of Rhed might reside somewhere in the remaining portion (i.e., within amino acids 371–478). Thus, we first investigated if the unknown functional region, ranging from 370 to 429 amino acids, might have an RNA-binding affinity. We deleted the 370–429 region from G478 and generated the G478del fragment (Fig. 1a). We similarly purified the G478del fragment as described for G478 (Fig. 2a). The purified G478del protein was indeed associated with hemin by showing a high absorbance value at 450 nm (Supplementary Fig. 2a). The estimated molecular mass of G478del was ~30.7 kDa, close to the size of the theoretical molecular mass of a dimer, ~30 kDa (Supplementary Fig. 2b). These data indicate that the 370–429 region is not required for dimerization or association with hemin. We then compared the RNA-binding affinity of G478del with 10L10_UGU and 10L10_noUGU using EMSAs, and showed that G478del interacted with 10L10_UGU (*K*_d_ = 5.73 ± 0.39 μM) with a higher affinity than it did with 10L10_noUGU (*K*_d_ = 16.8 ± 1.48 μM) (Fig. 2b, c). These suggest that the 370–429 region is dispensable for the UGU-recognition.

We further fragmented the G478del protein by deleting the 461–478 region and thus generated G460del (Fig. 2d). G460del lost its hemin-associating ability (Supplementary Fig. 2c), though it could still form dimers (Supplementary Fig. 2d). In addition, the EMSA results demonstrated that G460del dramatically lost its RNA-binding affinity (Fig. 2e, f). This suggests that amino acids 460–478 are critical for the hemin-association and RNA-interaction ability of Rhed, which might also include interaction with UGU.

### Amino acids 461–463 are responsible for UGU-interaction

**Fig. 3:**
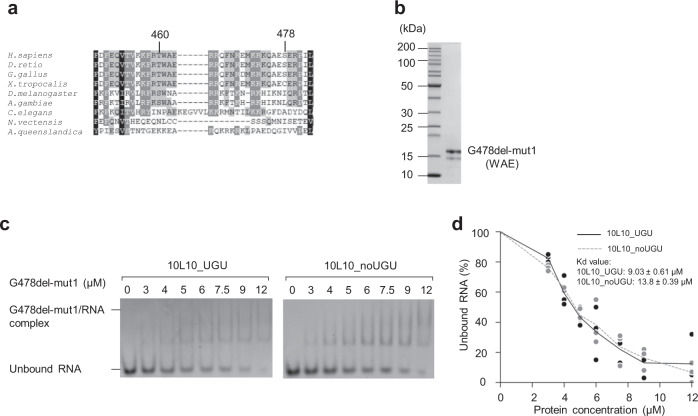
Amino acids 461–463 are responsible for recognizing the UGU motif. **a** Multiple sequence alignment of DGCR8 proteins from different organisms. The alignment method was as previously described^17^. The region from 448–483 of human (*H. sapiens*) DGCR8 is shown. *Homo sapiens* (*H. sapiens*), *Danio rerio* (*D. rerio*), *Gallus gallus* (*G. gallus*), *Xenopus tropicalis* (*X. tropicalis*), *Drosophila melanogaster* (*D. melanogaster*), *Anopheles gambiae* (*A. gambiae*), *Caenorhabditis elegans* (*C. elegans*), *Nematostella vectensis* (*N. vectensis*), and *Amphimedon queenslandica* (*A. queenslandica*). The black, dark gray, and light gray boxes represent 100, 80, and 60% sequence similarities, respectively. **b** The purified G478del-mut1 protein, which contains amino acids AGQ in the 461–463 region (instead of WAE, which are in the WT protein), was analyzed by SDS-PAGE. **c** The EMSAs for G478del-mut1. Various amounts of G478del-mut1 (ranging from 0 to 12 μM) were mixed with 1 μM 10L10_UGU or 10L10_noUGU RNA in a 10 μL reaction solution. **d** Quantification of the EMSA data for G478del-mut1 in (**c**). The density of each RNA band on the gel was measured using Image Lab 6.0 (Bio-Rad), and the results were obtained from three independent experiments.

We here reasoned that the amino acids recognizing UGU might be in the 460–478 region. As the UGU motif in pri-miRNAs is conserved from *Drosophila* to humans^8^, the amino acids, responsible for the UGU-recognition, might also be preserved in these different animal species. We then aligned the human (i.e., *Homo sapiens*) DGCR8 polypeptide with those from different organisms (i.e., *Danio rerio*, *Gallus gallus*, *Xenopus tropicalis*, *Drosophila melanogaster*, *Anopheles gambiae, Caenorhabditis elegans*, *Nematostella vectensis*, and *Amphimedon queenslandica*). We identified several amino acids in the 460–478 region that were conserved from *Drosophila* to humans (Fig. 3a). We then generated five different mutations of G478del (Supplementary Fig. 3a). The mutant G478del proteins were purified (Fig. 3b, Supplementary Fig. 3b) as described for G478del, and their RNA-binding affinity was estimated for both 10L10_UGU and 10L10_noUGU using the EMSAs (Supplementary Fig. 3c). The amino acids that are important for UGU-binding should satisfy three conditions: (1) The mutant protein, which has mutations in these amino acids, should maintain a dimeric form; (2) it should also associate with hemin; and (3) its RNA-binding affinity should be not much different for 10L10_UGU and 10L10_noUGU. Among the five mutant proteins generated, we found that the G478del-mut1 protein bound to 10L10_UGU and 10L10_noUGU quite similarly. In contrast, the other mutant G478del proteins showed a much weaker RNA-binding affinity for 10L10_noUGU (Supplementary Fig. 3c). We then quantified the RNA-binding affinity of G478del-mut1 with 10L10_UGU and 10L10_noUGU using the EMSAs. In the three experiments conducted, our data confirmed that G478del-mut1 only slightly discriminated between the two different substrates with or without UGU (*K*_d_ for 10L10_UGU: 9.03 ± 0.61 μM, *K*_d_ for 10L10_noUGU: 13.8 ± 0.39 μM, Fig. 3c, d). We also demonstrated that G478del-mut1 retained the same hemin-association and dimerization ability as G478del (Supplementary Fig. 3d, e). This indicates that amino acids 461–463 are essential for the UGU-recognition.

### Amino acids 461–463 are essential for pri-miRNA processing

**Fig. 4:**
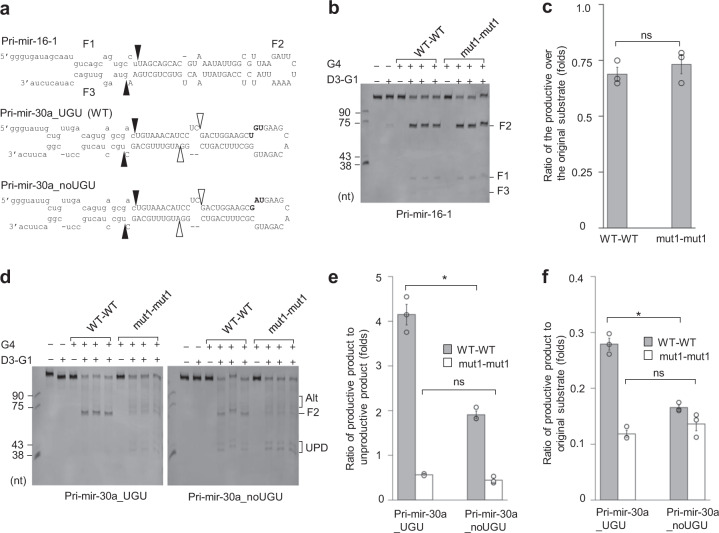
Amino acids 461–463 are responsible for the accuracy and efficiency of Microprocessor cleavage. **a** Diagrams of pri-mir-16-1, pri-mir-30a_UGU (WT), and pri-mir-30a_noUGU. The uppercase letters represent the pre-miRNA region. The solid black and white arrowheads indicate the productive and unproductive cleavage sites of Microprocessor, respectively. **b** The pri-mir-16-1 processing by Microprocessor. Pri-mir-16-1 (0.5 μM) was processed with D3–G1 (0.1 μM) and/or either G4 WT–WT (0.05 μM) or G4 mut1–mut1 (0.05 μM) for 60 min, as described in “Methods”. **c** Quantification of the data shown in (**b**). The density of each band was determined using Image Lab 6.0, and the ratio of the F2 product (pre-mir-16-1) to the original substrate (pri-mir-16-1) was calculated from three independent experiments. G4 WT–WT vs. G4 mut1–mut1: *p* = 0.350. **d** The pri-mir-30a_UGU and pri-mir-30a_noUGU processing by Microprocessor. Pri-mir-30a_UGU or pri-mir-30a_noUGU (0.5 μM) was processed by D3–G1 (0.2 μM) and/or either G4 WT–WT (0.1 μM) or G4 mut1–mut1 (0.1 μM) for 60 min, as described in “Methods”. Alt and UPD mean the alternative and unproductive cleavages, respectively. **e**, **f** Quantification of the data shown in (**d**). The ratio of the productive product (F2) to the unproductive product in (**e**), and the ratio of the productive product (F2) to the original substrate (pri-mir-30a_UGU or pri-mir-30a_noUGU) in (**f**), were calculated from three independent experiments. Productive product/unproductive product of pri-mir-30a_UGU vs. pri-mir-30a_noUGU for G4 WT–WT: *p* = 0.018 and G4 mut1–mut1: *p* = 0.154. Productive product/original substrate of pri-mir-30a_UGU vs. pri-mir-30a_noUGU for G4 WT–WT: *p* = 0.003 and G4 mut1–mut1: *p* = 0.182. The asterisks (*) and (ns) indicate statistically significant and nonsignificant differences, respectively, from the two-sided *t* test.

Next, we investigated the effects of amino acids 461–463 on the ability of Microprocessor to process pri-miRNAs. We generated a mut1 mutation in the G4 fragment (amino acids 285–750) of DGCR8 and purified it as described in “Methods” (Supplementary Fig. 3f). We also purified the D3 fragment (amino acids 390–1365) of DROSHA in a complex with the G1 fragment (amino acids 728–750) of DGCR8 as described in “Methods” (Supplementary Fig. 3f). It should be noted that G1 functions to stabilize and solubilize DROSHA. The Microprocessor complex was then reconstituted by mixing the G4 dimer with the D3–G1 complex. It has previously been shown that G1 can be efficiently replaced by the G4 dimer^10^. Here, we found that both G4 WT–WT (wild-type) and G4 mut1–mut1 could similarly stimulate DROSHA to cleave pri-mir-16-1, which lacked a UGU motif (Fig. 4a–c). However, G4 mut1–mut1 failed to increase the specific cleavage of DROSHA on pri-mir-30a, which contained a UGU motif (Fig. 4a, d–f). This suggests that G4 mut1–mut1 might lose its ability to interact with UGU. In addition, while G4 WT–WT showed a different cleavage activity between pri-mir-30a_UGU and noUGU, G4 mut1–mut1 exhibited a similar level of activity on both substrates. In other words, G4 mut1–mut1 could not distinguish between pri-mir-30a_UGU and pri-mir-30a_noUGU (Fig. 4d–f). These data support the conclusion that amino acids 461–463 are critical for the UGU-recognition, and they further emphasize the importance of the DGCR8–UGU interaction in pri-miRNA processing.

### Roles of amino acids 461–463 from one DGCR8 monomer

**Fig. 5:**
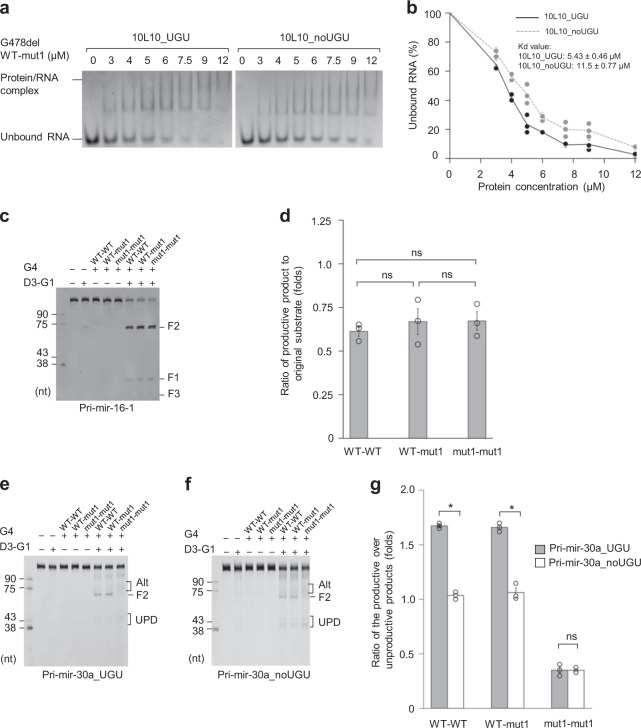
Amino acids 461–463 in just one DGCR8 monomer are necessary for the accuracy and efficiency of microprocessor cleavage. **a** EMSAs of the hybrid G478del WT–mut1 protein. Various amounts of G478del WT–mut1 (ranging from 0 to 12 μM) were mixed with 1 μM of either 10L10_UGU or 10L10_noUGU RNA in a 10 μL reaction solution. **b** Quantification of the EMSA data shown in (**a**). The density of each RNA band was determined using Image Lab 6.0 (Bio-Rad), and the results were obtained from three independent experiments. **c** The pri-mir-16-1 processing by microprocessor. Pri-mir-16-1 (0.5 μM) was processed by 0.1 μM D3–G1 and 0.05 μM G4 WT–WT, G4 WT–mut1, or G4 mut1–mut1 in the reaction buffer for 60 min, as described in “Methods”. **d** Quantification of the data shown in (**c**). The estimated ratio of the productive product (F2) to the original substrate was calculated from three independent experiments. G4 WT–WT vs. G4 WT–mut1: *p* = 0.534, G4 WT–mut1 vs. G4 mut1–mut1: *p* = 0.875, G4 WT–WT vs. G4 mut1–mut1: *p* = 0.373. **e**, **f** The pri-mir-30a_UGU (**e**) and pri-mir-30a_noUGU (**f**) processing by Microprocessor. Pri-mir-30a_UGU or pri-mir-30a_noUGU (both at 0.5 μM) was processed by 0.2 μM D3–G1 and 0.1 μM of G4 WT–WT, G4 WT–mut1, or G4 mut1–mut1 in the reaction buffer for 60 min, as described in “Methods”. Alt and UPD mean the alternative and unproductive cleavages, respectively. **g** Quantification of the data shown in (**e**, **f**). The ratio of the productive (F2) to unproductive products was calculated from three independent experiments. Pri-mir-30a_UGU vs. pri-mir-30a_noUGU for G4 WT–WT: *p* = 0.002, G4 WT–mut1: *p* = 0.002, and G4 mut1–mut1: *p* = 0.951. The asterisks (*) and (ns) indicate statistically significant and nonsignificant differences, respectively, from the two-sided *t* test.

Since the DGCR8 dimer recognizes and interacts with the UGU motif of pri-miRNA, we investigated the contribution of each monomer to the UGU-recognition. We purified a hybrid dimer containing one G478del–WT and one G478del-mut1 subunit. In brief, one kanamycin maker-containing plasmid and one ampicillin maker-containing plasmid, which expressed 10×His-tagged G478del-mut1 and protein G-tagged G478del–WT, respectively, were co-transformed into *E. coli* (Supplementary Fig. 4a). The transformed cells were cultured in a medium, supplemented with both kanamycin and ampicillin antibiotics, ensuring that the surviving cells obtained both plasmids. The G478del hybrid proteins were purified through two affinity columns, Ni^2+^-agarose and IgG–sepharose, which captured the G478del-mut1 and G478del–WT proteins, respectively. Therefore, when used in tandem, these two affinity columns allowed us only to collect the hybrid G478del WT–mut1 dimer (Supplementary Fig. 4b, c). An S-cation exchange column further purified the hybrid G478del WT–mut1 proteins (Supplementary Fig. 4b, c). We performed the EMSAs for the G478del WT–mut1 dimer, and found that it had a higher RNA-binding affinity to 10L10_UGU than to 10L10_noUGU RNA (Fig. 5a, b, *K*_d_ for 10L10_UGU: 5.43 ± 0.46 μM, *K*_d_ for 10L10_noUGU: 11.5 ± 0.77 μM). This suggests that amino acids 461–463 from just one monomer are necessary for the UGU-interaction.

Next, we co-transformed the kanamycin marker-containing plasmid expressing a 10×His-tagged G4–mut1 and the ampicillin marker-containing plasmid expressing protein G-tagged G4–WT in *E. coli* (Supplementary Fig. 4a). The hybrid G4 WT–mut1 dimer was purified using a similar approach described above for the hybrid G478del WT–mut1 dimer (Supplementary Fig. 4b, d). We then reconstituted the Microprocessor complexes by mixing DROSHA (D3–G1) with the different G4 dimers (i.e., WT–WT, WT–mut1, or mut1–mut1). The processing activities of the resulting complexes were tested with pri-mir-16-1 and pri-mir-30a. The three reconstituted Microprocessor complexes showed no difference in processing pri-mir-16-1 (Fig. 5c, d). However, they showed different processing activities for pri-mir-30a such that G4 WT–WT and WT–mut1 exhibited a similar specific activity, which was significantly higher than that of G4 mut1–mut1 (Fig. 5e–g). In EMSAs, the G4 mut1–mut1 dimer also showed a lower RNA-binding affinity for pri-mir-30a compared with G4 WT–WT and G4 WT–mut1 dimers, which exhibited a similar RNA-binding affinity (Supplementary Fig. 5a, b). In addition, G4 WT–WT and WT–mut1 could distinguish between pri-mir-30a_UGU and pri-mir-30a_noUGU, but mut1–mut1 could not (Fig. 5e–g). These results suggest that the G4 WT–mut1 dimer retains the comparable level of ability as the G4 WT–WT dimer to recognize and interact with the UGU motif. Therefore, these data indicate that amino acids 461–463 in just one DGCR8 monomer are necessary for the UGU-interaction.

### Amino acids 461–463 are critical for miRNA expression

**Fig. 6:**
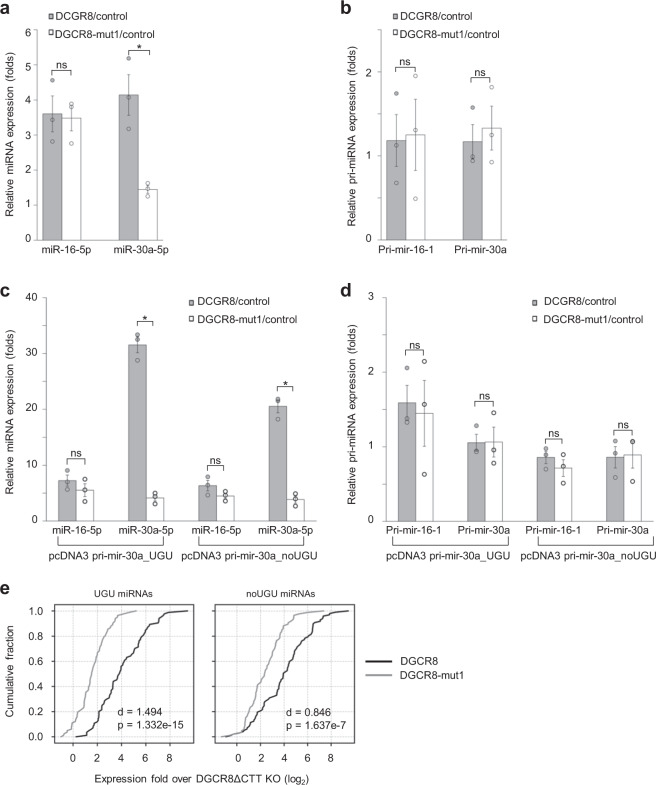
Amino acids 461–463 are critical for Microprocessor processing in human cells. **a** The DGCR8ΔCTT knockout (KO) cells were transfected with pCK (control), pCK-DGCR8–WT, or pCK-DGCR8–mut1. The miRNA expression of these transfected cells was estimated by qPCR as described in “Methods”. The results were obtained from three independent experiments (miR-16-5p DGCR8/control vs. miR-16-5p DGCR8–mut1/control: *p* = 0.857, miR-30a-5p DGCR8/control vs. miR-30a-5p DGCR8–mut1/control: *p* = 0.010). **b** The pri-miRNA expression in the transfected cells in (**a**) was estimated by qPCR and normalized against GAPDH. The results were obtained from three independent experiments (pri-mir-16-1 DGCR8/control vs. pri-mir-16-1 DGCR8–mut1/control: *p* = 0.904, pri-mir-30a DGCR8/control vs. pri-mir-30a DGCR8–mut1/control: *p* = 0.651). **c** The miR-30a expression in the DGCR8ΔCTT KO cells transfected with pCK-DGCR8–WT or mut1, pcDNA3-pri-mir-16-1, and pcDNA3-pri-mir-30a_UGU or pcDNA3-pri-mir-30a_noUGU. The results were obtained from three independent experiments (for the pcDNA3-pri-mir-30a_UGU transfected cells: miR-16-5p DGCR8–WT/control vs. miR-16-5p DGCR8–mut1/control: *p* = 0.322, miR-30a-5p DGCR8–WT/control vs. miR-30a-5p DGCR8–mut1/control: *p* = 5.5e−5; for the pcDNA3-pri-mir-30a_noUGU transfected cells: miR-16-5p DGCR8–WT/control vs. miR-16-5p DGCR8–mut1/control: *p* = 0.161, miR-30a-5p DGCR8–WT/control vs. miR-30a-5p DGCR8–mut1/control: *p* = 2.2e−4). **d** The expression of pri-miRNAs in the transfected cells described in (**c**). The pri-miRNA expression was estimated by qPCR and normalized using GAPDH. The results were obtained from three independent experiments (for the pcDNA3-pri-mir-30a_UGU transfected cells: pri-mir-16-1 DGCR8–WT/control vs. pri-mir-16-1 DGCR8–mut1/control: *p* = 0.792, pri-mir-30a DGCR8–WT/control vs. pri-mir-30a DGCR8–mut1/control: *p* = 0.968; for the pcDNA3-pri-mir-30a_noUGU transfected cells: pri-mir-16-1 DGCR8–WT/control vs. pri-mir-16-1 DGCR8–mut1/control: *p* = 0.364, pri-mir-30a DGCR8/control vs. pri-mir-30a DGCR8–mut1/control: *p* = 0.897. The asterisks (*) and (ns) indicate statistically significant and nonsignificant differences, respectively, from the two-sided *t* test. **e** The cumulative fraction of miRNA expression levels in the rescued DGCR8ΔCTT KO cells. The miRNAs were classified according to the presence of an apical UGU motif in pri-miRNAs as described in “Methods”. Cohenʼs *d* values were calculated between the miRNA expression levels induced by DGCR8–WT and DGCR8–mut1. The *p* values were calculated with the two-sided Wilcoxon rank-sum test.

To investigate the role of amino acids 461–463 in the cellular expression of miRNAs, we ectopically expressed DGCR8–WT or DGCR8–mut1 in DGCR8ΔCTT knockout (KO) cells, which were previously prepared from HCT116 cells^26^. Note that the DGCR8ΔCTT KO cells still expressed the N-terminal region of DGCR8, which lacks the CTT domain required for the DROSHA interaction (Supplementary Fig. 6a). The expressions of DGCR8–WT and mut1 were comparable in the three repeated transfections (Supplementary Fig. 6b). We used a quantitative polymerase chain reaction (qPCR) to measure the expression of UGU miRNAs (miR-30a-5p and miR-191-5p) and noUGU miRNAs (miR-16-5p and miR-125a-5p). DGCR8–WT and DGCR8–mut1 produced a similar level of miR-16-5p and miR-125a-5p expression but a different level of miR-30a-5p and miR-191-5p expression, such that DGCR8–mut1 induced a significantly lower level of miR-30a-5p and miR-191-5p expression compared with DGCR8–WT (Fig. 6a, Supplementary Fig. 6c). The expression of pri-mir-16-1, pri-mir-30a, pri-mir-191, or pri-mir-125a was not significantly different when comparing DGCR8–WT and DGCR8–mut1 (Fig. 6b, Supplementary Fig. 6d). These results were consistent with the in vitro data (Fig. 4b, d), which show that Microprocessor–WT and mut1 complexes both retained a similar level of pri-miRNA substrates after the reaction. These data suggest that the DGCR8–mut1 allele is defective in expressing UGU-containing miR-30a-5p and miR-191-5p, resulting in the differential level of cellular miRNA expression.

We also investigated the effect of amino acids 461–463 on miRNA expression from the same pri-mir-30a backbone with/without the UGU motif in human cells. The two pri-miRNA plasmids (pri-mir-30a_UGU and pri-mir-16-1, or pri-mir-30a_noUGU and pri-mir-16-1) were co-transfected with the DGCR8 plasmid expressing either DGCR8–WT or DGCR8–mut1 in the DGCR8ΔCTT KO cells. We found that DGCR8–WT or mut1 induced a similar ectopic expression of miR-16-5p when pri-mir-16-1 was co-expressed with pri-mir-30a_UGU or pri-mir-30a_noUGU (Fig. 6c). In contrast, DGCR8–WT expressed a higher level of miR-30a-5p from pri-mir-30a_UGU than pri-mir-30a_noUGU (Fig. 6c). Unlike DGCR8–WT, DGCR8–mut1 exhibited a similar level of miR-30a-5p from both pri-mir-30a_UGU and pri-mir-30a_noUGU. These data support the accumulating evidence for the importance of amino acids 461–463 in distinguishing UGU and noUGU pri-miRNAs, thereby inducing the differential expression of miRNAs. In addition, the expression of pri-mir-16-1 and pri-mir-30a was not significantly different for either DGCR8–WT or DGCR8–mut1 (Fig. 6d). These results are again consistent with the in vitro data showing that both microprocessor–WT and mut1 complexes retained a similar level of pri-miRNA substrates after the reaction (Fig. 4b, d).

To examine the functions of the identified amino acids (461–463) in the expression of cellular miRNAs on a genome-wide scale, we profiled the expression of DROSHA-dependent miRNAs by sequencing the miRNAs of the DGCR8ΔCTT KO cells transfected with either the DGCR8–WT or mut1 plasmid. We found that DGCR8–mut1 induced a much lower level of these miRNAs expression than DGCR8–WT (Fig. 6e, Supplementary Fig. 6e, Supplementary Data 1). In addition, DGCR8–WT and DGCR8–mut1 showed a higher difference in expressing UGU miRNAs (Cohenʼs *d* = 1.494) than noUGU miRNAs (Cohenʼs *d* = 0.846) (Fig. 6e). This demonstrates the importance of amino acids 461–463 for cellular miRNA expression, especially for UGU miRNAs.

## Discussion

Rhed is a multi-functional domain of DGCR8, with dimerization, hemin association, and RNA-interacting properties^11,26,33–35^. These characteristics all contribute to the enzymatic mechanism of pri-miRNA processing by Microprocessor. It has previously been reported that the interaction between DGCR8 and the conserved UGU motif is critical for the enzymatic activity of human Microprocessor^10,26^. In this study, we demonstrated that Rhed is also responsible for recognizing the UGU motif, and we showed that three conserved amino acids 461–463, are essential for the UGU–DGCR8 interaction. We showed that the UGU–DGCR8 interaction was important for the expression of UGU-containing miRNAs in human cells by mutating these amino acids. Therefore, our study suggests that any mechanism that affects the UGU–DGCR8 interaction via these amino acids might alter the cellular expression of miRNAs. Potential mechanisms include protein modifications on these amino acids or protein–protein interactions that might either weaken or strengthen the interaction between DGCR8 and the UGU motif. For example, it is reported that in mice, RNA polymerase II negatively affects the expression of UGU-containing miRNAs, which therefore induces the differential expression of miRNAs^36^. It would be interesting to test if a similar mechanism might happen in human cells, and if RNA polymerase II might affect UGU–DGCR8 interaction via the amino acids 461–463.

DGCR8 is a dimer in Microprocessor^10,11,17^. However, our study suggests that amino acids 461–463 in just one of the two DGCR8 monomers are necessary for the UGU–DGCR8 interaction, thus effective and accurate pri-miRNA processing. However, it is known that (unlike the dimer), monomeric DGCR8 does not efficiently induce the accuracy and efficiency of cleavage on UGU-pri-miRNA^10,24,26^. This suggests that the UGU-binding site of DGCR8 might only be adequately folded in the dimer. This might explain why DGCR8 exists as a dimer, while there is a UGU motif in pri-miRNAs. Thus, we propose two possibilities: (1) the two monomers in the DGCR8 dimer form a single UGU-binding site. (2) Each monomer in the DGCR8 dimer possesses one UGU-binding site, and the UGU motif might interact with either UGU-binding site at a time. In the previous study, hemin appeared to strengthen the interaction between DGCR8 and the UGU motif^26^. Therefore, it is likely that hemin might affect the conformation of the Rhed domain, including amino acids 461–463, so it can form a proper structure to interact with the UGU motif. Future DGCR8–RNA structure studies might clarify these proposed models.

## Methods

### Purification of the D3–G1 complex

The pXab–D3 containing DROSHA fragment (D3, amino acids 390–1365), which was fused to protein G, and the pXC–G1 containing DGCR8 fragment (G1, amino acids 728–750), which was fused to CFP and the 10×His-tag at its C-terminus were transfected together into 200 dishes (100 mm in diameter) of HEK293E cells^10^. The transfected cells were collected after 3 days, and the cell pellets were dissolved in 100 ml T150 buffer (20 mM Tris-HCl (pH 7.5), 150 mM NaCl, and 2 mM β-mercaptoethanol), added with 2 μg/ml RNase A and protease inhibitor cocktail. The cell mixture was sonicated and centrifuged, after which the clear lysate portion was loaded onto a Ni^2+^-agarose column. The Ni^2+^-agarose column was then washed with 100 ml T150 plus 40 mM imidazole, and eluted with 100 ml T150 plus 200 mM imidazole. The peak fractions containing the recombinant proteins were mixed with a Q-sepharose column. This Q-sepharose column was then washed with T150 and eluted with T500 (20 mM Tris-HCl (pH 7.5), 500 mM NaCl, and 1 mM DTT). The eluted proteins were concentrated with a 100 kDa molecular weight cut-off (MWCO) centricon to obtain approximately 10–15 mg/ml. The concentrated proteins were loaded onto a HiLoad 16/600 Superdex 200 pg column (GE Healthcare). The peak fractions were collected and frozen in liquid nitrogen before being stored at −80 °C.

### Production and purification of DGCR8 fragments

To purify the G4 and G4-variant proteins, we used the basic T150 buffer recipe but adjusted the NaCl concentration. Thus, T50, T250, T300, and T2000 buffers contained 50, 250, 300, 2000 mM NaCl, respectively. The pET-28a vector containing the G4 fragment fused to an N-terminal GFP tag and a 10×His-tag^10^ was transformed into *E. coli* BL21(DE3)-CodonPlus-RIPL cells and protein production was induced by treatment with isopropyl β-d-1-thiogalactopyranoside (IPTG) at 30 °C overnight. A cell lysate was prepared in a T500 buffer and loaded onto a Ni^2+^-agarose column. The column was washed with T500 containing 20 mM imidazole and eluted with T500 containing 200 mM imidazole. The eluate was diluted with the buffer containing 20 mM Tris-HCl (pH 7.5) and 1 mM DTT to obtain a final 150 mM NaCl buffer and was loaded onto an SP–sepharose column (GE Healthcare). The column was washed with T150 and eluted with T500 containing 1 mM DTT, after which the eluate was concentrated using a 50 kDa MWCO centricon and then loaded onto a HiLoad 16/600 Superdex 75 pg column (GE Healthcare). The peak fractions were again collected and frozen in liquid nitrogen before storage at −80 °C.

The G478 and its variants were purified as described for the G4 fragments. However, the purified proteins in the eluate from the SP–sepharose column were concentrated using a 30 kDa MWCO centricon, and then they were loaded onto a Superdex 200 pg 10/300 column (GE Healthcare). Again, the peak fractions collected were frozen in liquid nitrogen and stored at −80 °C.

### Purification of the DGCR8 WT–mut1 dimer

A plasmid with a kanamycin marker expressing a 10×His-tagged G4–mut1 or 10×His-tagged G478del-mut1 and a plasmid with ampicillin marker expressing protein G-tagged G4–WT (wild-type) or protein G-tagged G478del–WT were co-transformed into *E. coli* BL21(DE3)-CodonPlus-RIPL cells. A single colony was selected and seeded into LB medium containing both kanamycin and ampicillin to ensure that the transformed cells contained both plasmids. Protein expression was induced by treatment with 0.2 mM IPTG overnight at 30 °C. Protein dimers consisting of one mut1 monomer and one WT monomer were selectively purified through two affinity columns, one Ni^2+^-agarose column and one IgG–sepharose column. Specifically, the Ni^2+^-agarose column captured proteins containing one or two mut1 monomers (i.e., G4–mut1 or G478–mut1) with a His-tag. The eluted proteins from the Ni^2+^-agarose column were then bound to the IgG–sepharose column, which interacted only with WT proteins (G4 or G478del) containing protein G. The IgG–sepharose-bound proteins were treated with human Rhinovirus 3C proteases at 4 °C overnight. As a result, G4 WT–mut1 or G478del WT–mut1 dimers were released and then eluted from the IgG–sepharose. An S-cation exchange column further purified the dimeric proteins prior to the enzymology assays and EMSAs.

### Preparation of the RNA substrates

DNA templates used in in vitro transcription (IVT) for synthesizing RNA substrates were produced by PCR. The templates and primer pairs used for PCR were shown in Supplementary Table 1. The DNA templates (200 ng) were incubated in a 20 μL IVT mixture, provided in the MEGAscript T7 Kit, for 10 h at 37 °C. The IVT reaction was terminated by adding a 20 μL TBE–urea sample buffer. The mixture was loaded onto 10% urea–polyacrylamide gel electrophoresis (PAGE) and the gel was stained with ethidium bromide. The RNAs were cut from gel under UV light and gel-purified. The purified RNAs were finally measured by a nanodrop spectrophotometer and reloaded onto 10% urea–PAGE for confirming their integrity.

### Pri-miRNA processing assay

The pri-miRNA processing assay was conducted at 37 °C in 10 μL of the reaction buffer containing 50 mM Tris-HCl (pH 7.5), 150 mM NaCl, 2 mM MgCl_2_, 1 mM DTT, 0.2 μg/μL bovine serum albumin (BSA) and 10% glycerol. The concentrations of RNA substrates and enzymes were shown in the figure legends. The reaction was terminated by adding a 10 μL TBE–urea sample buffer and was immediately cooled on ice. The reaction mixture was further incubated with 20 μg Proteinase K for 30 min at 37 °C followed by 30 min at 50 °C. Finally, the reaction mixture was denatured at 95 °C for 10 min and then quickly chilled on ice before being loaded onto a 10% urea–PAGE gel with size markers.

### Electrophoresis mobility shift assay

Different concentrations of the DGCR8 proteins were incubated with 10 pmol RNA in 10 μL Tris-HCl buffer (containing 50 mM Tris-HCl (pH 7.5), 250 mM NaCl, 10% glycerol, 0.2 mg/ml BSA, 1 mM DTT and 2 mM EDTA). The EMSA reaction was carried out on ice for 60 min. Ten microlitre of each sample was then loaded onto a 4% native PAGE gel and ran at 4 °C for 45 min. The gel was then stained with ethidium bromide, and images were acquired with the Bio-Rad Gel Doc XR + system. The RNA densities in the gels were determined using Image Lab (Bio-Rad) software version 6.0. Nonlinear regression curves for the RNA-binding and *K*_d_ values were calculated by using GraphPad Prism software version 8.3.1^37^.

### Rescue experiments and qPCR

The DGCR8ΔCTT KO cells were cultured in McCoyʼs 5A medium supplemented with 10% fetal bovine serum (FBS) in 60-mm dishes. When the cells achieved 20% confluence, they were transfected with 5 μg pCK, pCK-DGCR8, or pCK-DGCR8–mut1 plasmid using Lipofectamine 3000 (Invitrogen). After 2 days, the total RNA was isolated from the transfected cells using TRIzol Reagent. Totally, 50 ng of total RNA was used in the qPCR experiments for miRNAs, using TaqMan^™^ MicroRNA Reverse Transcription Kit (for miR-16-5p, miR-30a-5p, and U6) or the stem–loop reverse transcription primers (for miR-191-5p, miR-125a-5p, and U6), which were designed for each miRNA according to the method previously described^38^. The cDNAs used for the qPCR of pri-mir-16-1, pri-mir-30a, pri-mir-191, pri-mir-125a were synthesized from 1 μg total RNA using reverse primers designed for each pri-miRNA. The qPCR for miR-16-5p and miR-30a-5p was performed using TaqMan^™^ MicroRNA Assay (PN4427975) for miR-16-5p (000391) and miR-30a-5p (000417), and was normalized to that of U6 (001973). The qPCR for miR-191-5p, miR-125a-5p, and all pri-miRNAs was carried out using the SYBR^®^ Green master mix (Bio-Rad). The primer sequences were shown in Supplementary Table 1. The DGCR8ΔCTT KO and HEK293E cells were received from Narry Kimʼs lab (Seoul National University).

### Small RNA sequencing analysis

The total RNAs were isolated from the DGCR8ΔCTT KO cells, which were transfected with pCK, pCK-DGCR8–WT, or pCK-DGCR8–mut1. Next, the small RNA sequencing libraries were generated from these total RNAs using the TruSeq Small RNA Library Prep Kits. The adapter sequences were trimmed from the raw reads using cutadapt (-a AGATCGGAAGAG CACACGTC -A GATCGTCGGACTGTAGAACTCTGAAC)^39^. The trimmed paired-end reads were then joined using fastq-join (default parameters)^40^. Subsequently, the joined reads, which ranged from 18 to 26 nt, were selected. The low-quality reads were further removed (minimum quality score: 20, minimum percent of bases having the quality: 90) using FASTX-Toolkit (http://hannonlab.cshl.edu/fastx_toolkit/index.html). The selected high-quality reads were then mapped to the human genome (hg38) using Bowtie2^41^. The reads, which were mapped to miRNA loci (using the miRNA annotation obtained from miRBase v22; miRbase.org) and showed unambiguous alignments with no mismatches except for those at the 3′-ends, were collected for subsequent analysis. The read counts of miRNAs were converted to reads per million (rpm) and then normalized to the geometric mean of hsa-mir-320a and hsa-mir-320b, which were DROSHA-independent miRNAs. The normalized values were averaged for the three biological replicates. We classified DROSHA-dependent miRNAs obtained from MirGeneDB 2.0^42^ into UGU or noUGU groups. The expression fold changes over DGCR8ΔCTT KO (log2) from rescue experiments were shown in Supplementary Data 1.

### Statistics and reproducibility

The statistical tests were conducted for those experiments, which were repeated three times. Quantitative data were shown as the mean ± s.e.m. We estimated *p* values for qPCR experiments and cleavage assays using the two-sided *t* test, and for small RNA sequencing experiments using a two-sided Wilcoxon rank-sum test.

### Reporting summary

Further information on research design is available in the Nature Research Reporting Summary linked to this article.

## Supplementary information

Supplementary Information

Description of Additional Supplementary Files

Supplementary Data 1

Supplementary Data 2

Reporting Summary

## Data Availability

RNA sequencing data were deposited to the Gene Expression Omnibus (GEO: GSE140209). The source data underlying Figs. 1d, e, 2b, c, e, f, 3c, d, 4b, c, d–f, 5a–g, and 6a–e are provided as a Source Data file. The information and data from this paper are available from the corresponding author upon reasonable request.
