# Supplementary material for: Select amino acids in DGCR8 are essential for the UGU-pri-miRNA interaction and processing
Source: Commun Biol. 2020 Jul 3;3:344. doi: 10.1038/s42003-020-1071-5 (PMC7334207; doi:10.1038/s42003-020-1071-5)
Supplement: Supplementary file 1 — Supplementary Information [file 42003_2020_1071_MOESM1_ESM.pdf]

Figure S1

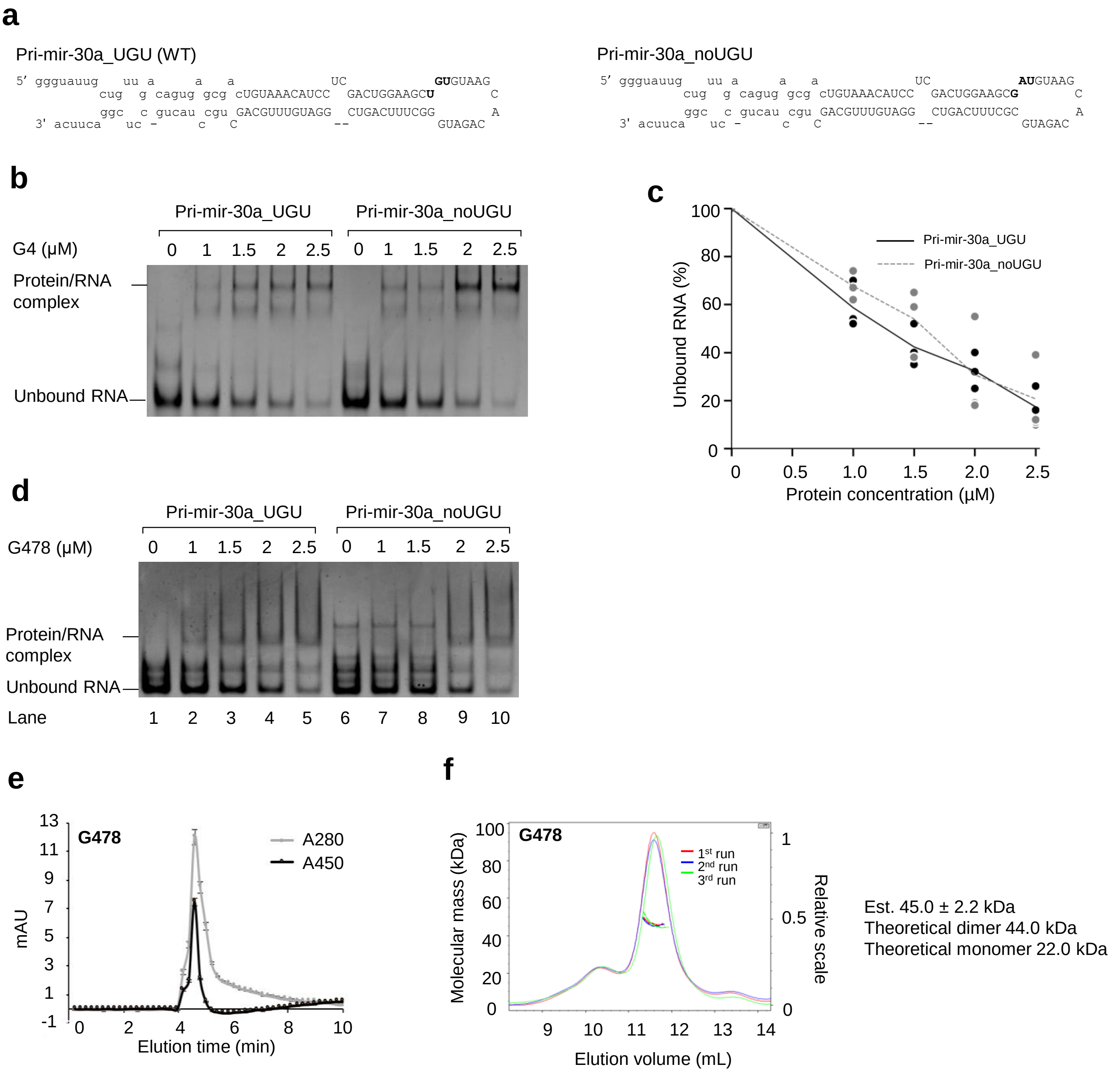

Supplementary Figure 1. Purification of the DGCR8 fragments. **a** Diagram of pri-mir-30a_UGU (WT) and pri-mir-30a_noUGU. The capital letters indicate the pre-miRNA region. **b** The EMSAs for G4 protein. Various amounts of G4 (ranging from 0 µM to 2.5 µM) were mixed with 1 µM of pri-mir-30a_UGU or pri-mir-30a_noUGU in a 10 µL reaction solution. **c** Quantification of the EMSA data shown in C. The density of each RNA band was determined using Image Lab 6.0 (Bio-Rad), and the results were obtained from three independent experiments. **d** The EMSAs for G478 protein. Various amounts of G478 (ranging from 0 µM to 2.5 µM) were mixed with 1 µM of pri-mir-30a_UGU or pri-mir-30a_noUGU in a 10 µL reaction solution. **e** The high-performance liquid chromatography profiles of the purified G478 proteins. Five µg of the purified proteins were loaded onto a Superdex 75 Increase 5/150 GL column (GE Healthcare) run with the Agilent 1260 Infinity II Quat LC system. The proteins were monitored at wavelengths of 280 nm and 450 nm for the levels of protein and hemin, respectively. The high correlation between the 450 nm and 280 nm measurements, indicates the association between the purified proteins and hemin. **f** The estimated molecular mass of the purified G478 proteins using a Multi-Angle Light Scattering Detector (miniDAWN TREOS, Wyatt Technology Corporation). The estimated molecular mass of each protein was obtained from three repeated experiments. The theoretical molecular mass was estimated based on the number of amino acids in each protein. Estimated (Est.) and theoretical indicate the estimated and theoretical molecular masses, respectively.

Figure S2

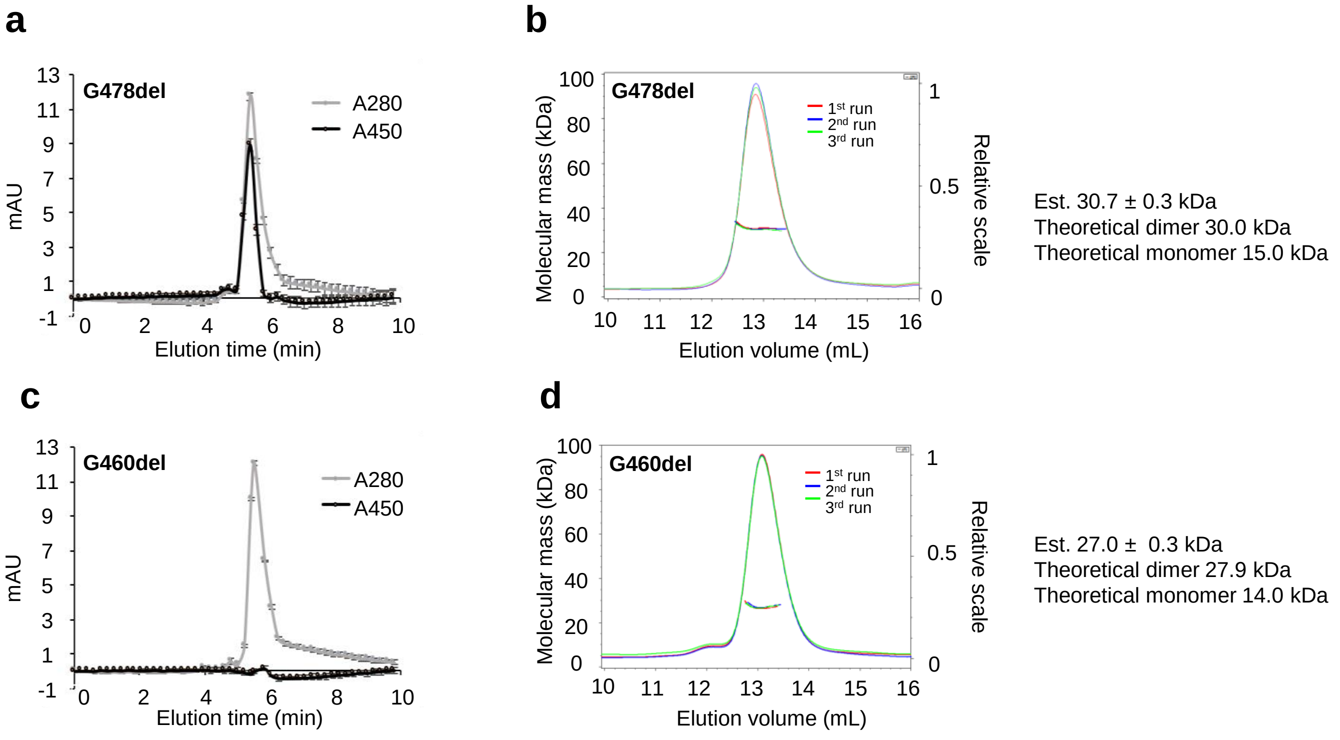

Supplementary Figure 2. The dimeric state and hemin level in the purified proteins. a, c The high-performance liquid chromatography profiles of the purified G478del and G460del proteins were collected as described in Supplementary Fig. 1e. **b, d** The estimated molecular mass of the purified G478del and G460del proteins were carried out as described in Supplementary Fig. 1f.

Figure S3

a

Mutant name	mut0	mut1	mut2	mut3	mut4
Wild-type	458FR459	461WAE463	464RR465	467FNR469	472KRK474
Mutation	458AA459	461AGQ463	464AA465	467ADA469	472AAA474

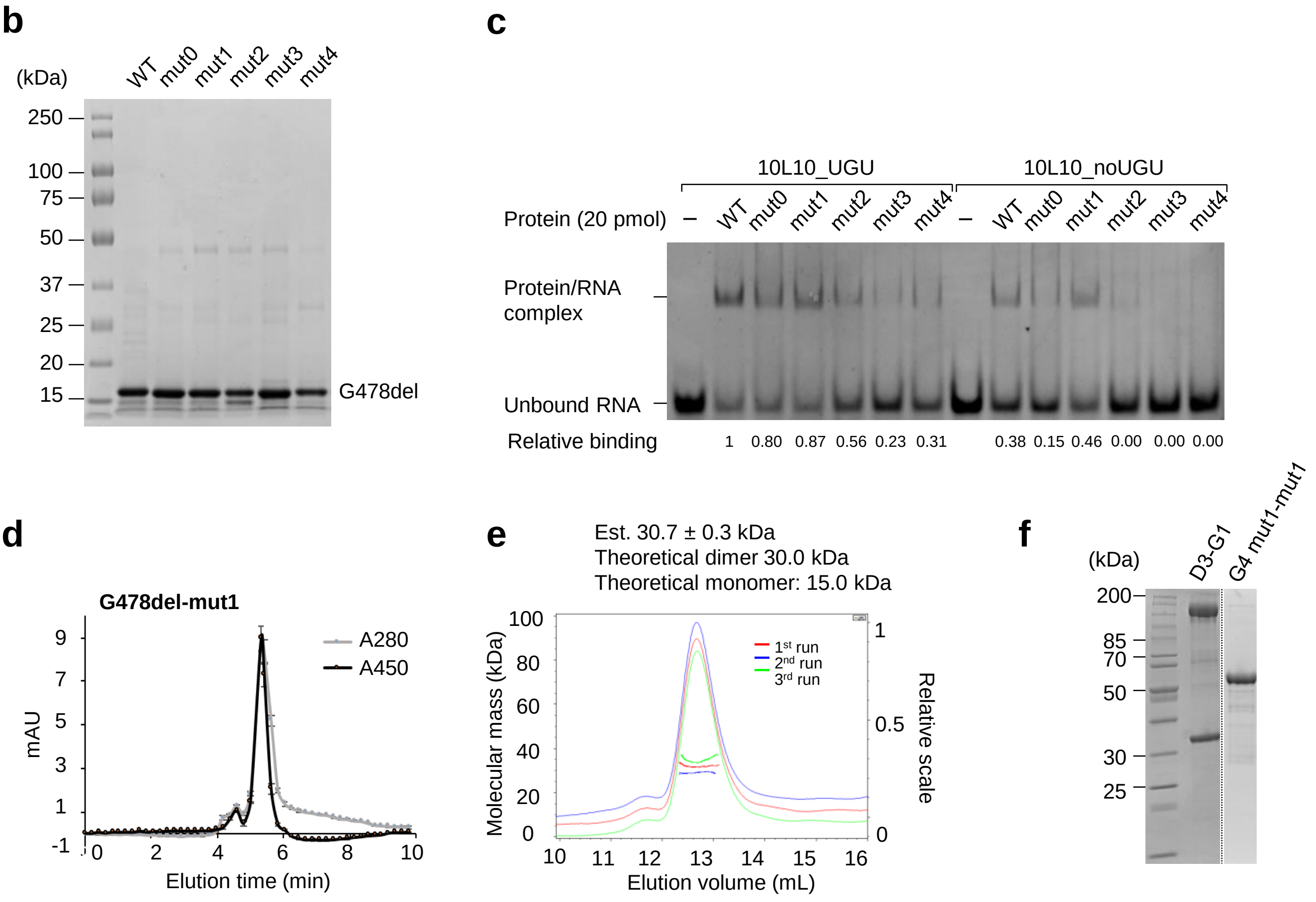

Supplementary Figure 3. Amino acids 461–463 are essential for UGU-recognition. a Table to show the amino acids of the wild-type (WT) and mutant G478del proteins. **b** The purified WT and mutant G478del proteins analyzed by SDS-PAGE. **c** The EMSAs for the G478del mutant proteins. G478del (20 pmol) was mixed with 10 pmol of either 10L10_UGU or 10L10_noUGU. The reaction mixture was run on a 4% of native PAGE. **d** High-performance liquid chromatography profiles of the G478del-mut1 proteins were obtained as described in Supplementary Fig. 1e. **e** The molecular mass of the purified G478del-mut1 proteins was estimated as described in Supplementary Fig. 1f. **f** The purified D3-G1 and G4 mut1-mut1 proteins were analyzed by SDS-PAGE.

Figure S4

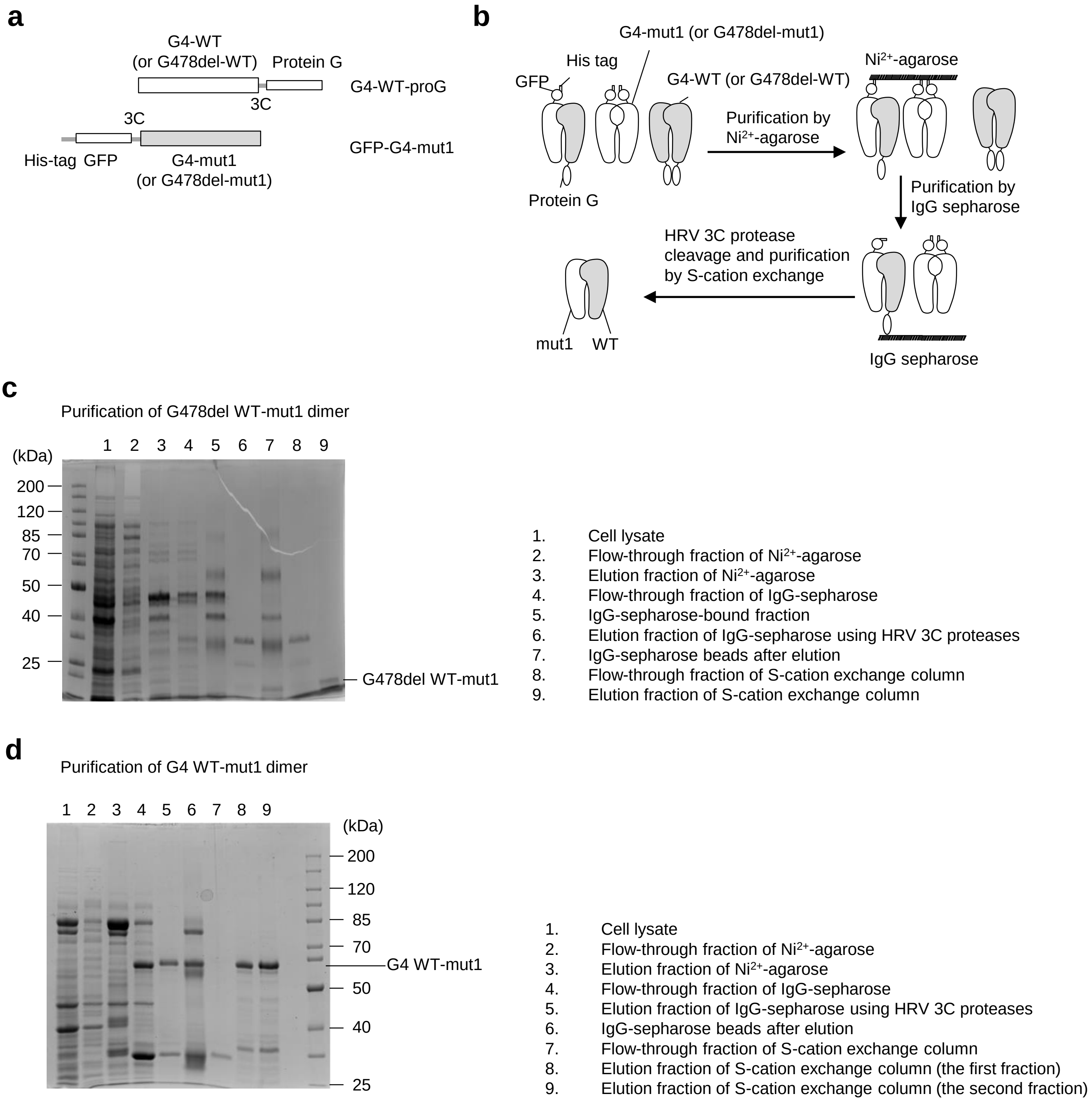

Supplementary Figure 4. Amino acids 461–463 in just one of the DGCR8 monomers, are necessary to induce the accuracy and efficiency of Microprocessor cleavage. **a** Protein constructs of G4-WT, G478del-WT, G4-mut1, and G478-mut1. G4-WT or G478del-WT was tagged with protein G at their C-terminus in the ampicillin marker-containing plasmid. G4-mut1 or G478del-mut1 was tagged with the His-tag and GFP at their N-terminus in the kanamycin marker-containing plasmid. 3C indicates the HRV 3C protease cleavage site. **b** Diagram illustrating the protein purification methodology used for the hybrid WT-mut1 dimer. **c, d** The resulting proteins (i.e., G478del WT-mut1 in (**c**), and G4 WT-mut1 in (**d**) from every step of the hybrid WT-mut1 dimer purification were analyzed by SDS-PAGE.

Figure S5

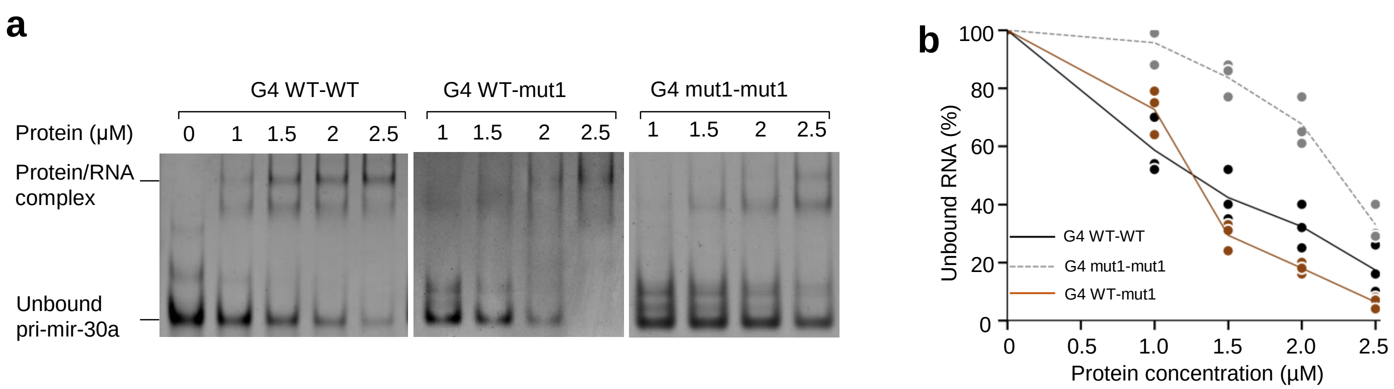

Supplementary Figure 5. Amino acids 461–463 in just one DGCR8 monomer are necessary for the accuracy and efficiency of Microprocessor cleavage. **a** The EMSAs of the hybrid G4 WT-mut1 protein. Various amounts of G4 WT-WT, WT-mut1, and mut1-mut1 (ranging from 1 μM to 2.5 μM) were mixed with 1 μM of pri-mir-30a in a 10 μL reaction solution. **b** Quantification of the EMSA data shown in (**a**). The density of each RNA band was determined using Image Lab 6.0 (Bio-Rad), and the results were obtained from three independent experiments.

Figure S6

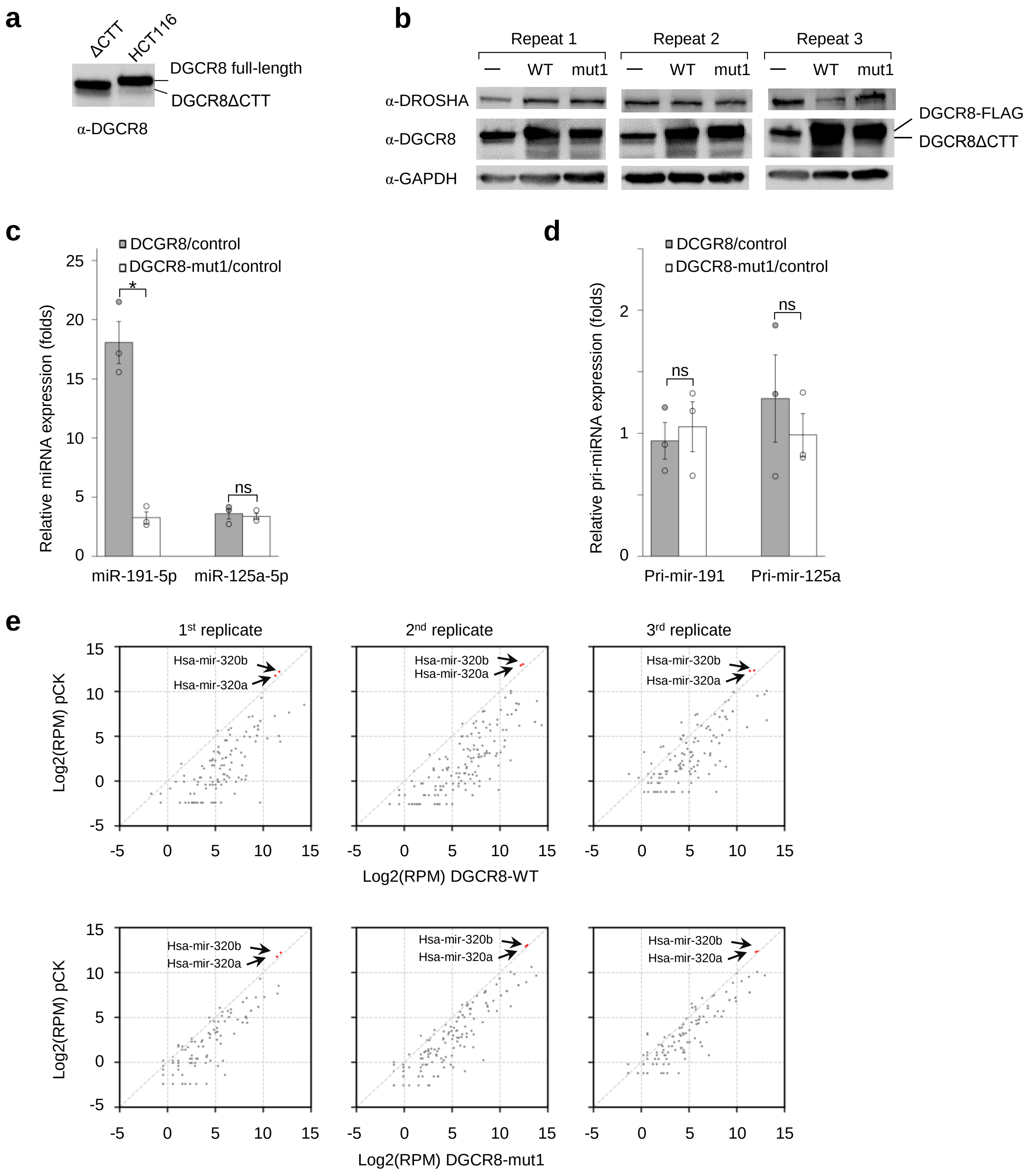

a
ΔCTT
HCT116
DGCR8 full-length
DGCR8ΔCTT
α-DGCR8
b
Repeat 1
Repeat 2
Repeat 3
— WT mut1
α-DROSHA
α-DGCR8
α-GAPDH
DGCR8-FLAG
DGCR8ΔCTT
c
DCGR8/control
DGCR8-mut1/control
Relative miRNA expression (folds)
miR-191-5p
miR-125a-5p
ns
d
DCGR8/control
DGCR8-mut1/control
Relative pri-miRNA expression (folds)
Pri-mir-191
Pri-mir-125a
ns
e
1st replicate
2nd replicate
3rd replicate
Hsa-mir-320b
Hsa-mir-320a
Log2(RPM) pCK
Log2(RPM) DGCR8-WT
Log2(RPM) DGCR8-mut1

Supplementary Figure 6. Amino acids 461–463 are critical for Microprocessor processing in human cells. **a** The DGCR8 full-length and DGCR8ΔCTT proteins in HCT116 and DGCR8ΔCTT knockout (KO) cells, respectively, were confirmed by western blot usin α-DGCR8 antibody (4D4). **b** The confirmation of the DGCR8 overexpression in the DGCR8ΔCTT KO cells, which were transfected with the pCK, pCK-DGCR8-WT, or pCK-DGCR8-mut1. The transfected cells were lysed with the lysis buffer, and western blot were carried out with 60004-1-lg (Proteintech) for GAPDH, α-DGCR8 antibody (4D4) for DGCR8, and α-DROSHA antibody (21D3) for DROSHA. Both α-DROSHA and α-DGCR8 antibodies were gifts from Dr. Narry Kim's lab, Seoul National University. **c** Expression of miRNA after the rescue. DGCR8ΔCTT KO cells were transfected with pCK (control), pCK-DGCR8-WT or pCK-DGCR8-mut1. The expression of miRNA in these transfected cells was estimated for miR-191-5p and miR-125a-5p by qPCR using the primers shown in Supplementary Table 1, using U6 as a normalization factor. The results were obtained from three independent experiments (miR-191-5p DGCR8-WT/control vs. miR-191-5p DGCR8-mut1/control: $p = 0.001$, miR-125a-5p DGCR8-WT/control vs. miR-125a-5p DGCR8-mut1/control: $p = 0.680$). **d** The expression of pri-miRNAs in the transferred DGCR8ΔCTT KO cells in (**c**). The expression levels of pri-mir-191 and pri-mir-125a were estimated by qPCR and normalized against GAPDH. The results were obtained from three independent experiments. Pri-mir-191 DGCR8-WT/control vs. pri-mir-191 DGCR8-mut1/control: $p = 0.670$, pri-mir-125a DGCR8-WT/control vs. pri-mir-125a DGCR8-mut1/control: $p = 0.495$. The qPCR primers were presented in Supplementary Table 1. The asterisks (*) and (ns) indicate statistically significant and nonsignificant differences, respectively, from the two-sided t-test. **e** Global expression of DROSHA-dependent miRNAs, hsa-mir-320a and hsa-mir-320b, in the DGCR8ΔCTT KO cells, which were transfected with the pCK, pCK-DGCR8-WT, or pCK-DGCR8-mut1.

**Primer name**	**Sequence**	**Experiment**
miR-191-5p RT	GTCGTATCCAGTGCAGGGTCCGAGGTATTCGCACTGGATACGACCAG CTG	Reverse transcription primers for miRNAs
miR-125a-5p RT	GTCGTATCCAGTGCAGGGTCCGAGGTATTCGCACTGGATACGACTCA CAG	
Common miRNA Reverse	GTG CAG GGT CCG AGG T	qPCR primers for miRNAs
miR-191-5p Forward	GCCAACGGAATCCCAAAA	
miR-125a-5p Forward	GCTCCCTGAGACCCTTTA	
GAPDH Forward	ACCCACTCCTCCACCTTTGAC	qPCR primers for GAPDH
GAPDH Reverse	CCAGGGGTCTTACTCCTTGGAG	
F-T7-mir-16-1	TAATACGACTCACTATAGGG TGATAGCAATGTCAGCAGTTTAG	qPCR primers for pri-miRNAs
R-16-1	TAGAGTATGGTCAACCTTACTTCAGC	
F-T7-mir-30a	TAATACGACTCACTATAGGGTATTGCTGTTGACAGTGAGC	
R-30a	TGAAGTCCGAGGCAGTAGGCAGCTGC	

Supplementary Table 1. RT-qPCR primers
