# Supplementary material for: Select amino acids in DGCR8 are essential for the UGU-pri-miRNA interaction and processing
Source: Commun Biol. 2020 Jul 3;3:344. doi: 10.1038/s42003-020-1071-5 (PMC7334207; doi:10.1038/s42003-020-1071-5)
Supplement: Supplementary file 2 — Description of Additional Supplementary Files [file 42003_2020_1071_MOESM2_ESM.pdf]

Description of Additional Supplementary Files

File Name: **Supplementary Data 1**
Description: The microRNA profiles in transfected cells. DROSHA-dependent miRNAs were referred from MirgeneDB 2.0. The reads per million (rpm) of miRNAs were normalized to the geometric mean of DROSHA-independent miRNAs, hsa-mir-320a, and hsa-mir-320b. The average of the normalized values was calculated from the three biological replicates. UGU and noUGU miRNAs are derived from pri-miRNAs containing UGU and noUGU, respectively.

File Name: **Supplementary Data 2**
Description: Source Data file
